# Detection of Human and Fish Viruses in Marine Gastropods

**DOI:** 10.3390/ani12162122

**Published:** 2022-08-18

**Authors:** Francesca Errani, Sara Ciulli, Luciana Mandrioli, Patrizia Serratore, Enrico Volpe

**Affiliations:** Department of Veterinary Medical Sciences, Alma Mater Studiorum University of Bologna, 47042 Cesenatico, Italy

**Keywords:** gastropods, hepatitis A virus, histology, molluscs, molecular investigation, nervous necrosis virus, norovirus, *Tritia mutabilis*

## Abstract

**Simple Summary:**

Mollusca is one of the largest phyla in the animal kingdom that includes more than 100,000 existing species living in aquatic and terrestrial habitats. Within this phylum, marine molluscs are considered an important resource for fisheries, and gastropods represent 2% of marine molluscs fished worldwide. Similar to bivalves, gastropods are susceptible to environmental contamination, and they are able to accumulate microorganisms. However, despite their economic importance, only few studies have focused on the monitoring of viral contamination in their tissues and their possible role as carriers. In this study, the presence of human pathogenic viruses such as hepatitis A virus, but not noroviruses, different to the situation in bivalve molluscs, was found in gastropods. This finding suggests a low risk of food-borne viral infections for gastropod consumers. Furthermore, one of the most impactful pathogens for marine aquaculture, nervous necrosis virus (NNV), was detected in gastropods. However, the animal tissues examined did not show any histological changes, suggesting the absence of a pathogenic effect of NNV in the analyzed gastropods.

**Abstract:**

Marine gastropods represent a major food source for higher trophic levels and an important source of animal protein for humans. Like bivalve molluscs, gastropods can accumulate several types of contaminants; however, the bioaccumulation of microorganisms, particularly viruses, has been poorly investigated in these animals. This study focused on gastropods (*Tritia mutabilis*, *Bolinus brandaris* and *Rapana venosa*) collected during the fishing season from 2017 to 2021 in the north-western Adriatic Sea, and on clams (*Ruditapes philippinarum*) harvested in the same geographical area, in order to evaluate the presence of human and fish viruses in their tissues. A virological investigation was carried out on the digestive gland using molecular methods. The presence of hepatitis A virus was detected in one sample, whereas noroviruses were not present in the investigated specimens. Regarding fish viruses, it was possible to detect the presence of nervous necrosis virus (NNV) in 26.5% of the analyzed gastropods; however, the histological examination did not show any pathological changes in the nervous tissue in both NNV-positive and -negative batches. As a whole, the investigated gastropods showed the ability to bioaccumulate viruses; however, lower contamination by human viruses compared to bivalve molluscs was pointed out, posing a minor concern to human health.

## 1. Introduction

Gastropoda is the most diverse molluscan class that includes more than 75,000 existing species. Gastropods are highly abundant in marine ecosystems, playing an important ecological role as grazers, scavengers and predators. They also represent a major food source for higher trophic levels [1].

Furthermore, marine gastropods are an important source of animal protein for humans [1], and some gastropod species, such as *Tritia mutabilis*, represent an important resource of local income for small-scale fisheries in the Adriatic Sea [2].

Similar to bivalves, gastropods are very susceptible to environmental contaminants and have been shown to accumulate metals and marine biotoxins [1]. Furthermore, bioaccumulation of several pathogenic microorganisms has been reported in various mollusc species. In particular, studies focused on bivalve molluscs such as oysters, clams and mussels demonstrated how these animals are able to concentrate viruses even 100–1000 times more than the surrounding environment due to their filter-feeding activity [3].

Because of their ability to bioaccumulate microorganisms, these species may play an important role in the transmission of human pathogenic viruses such as noroviruses (NoVs) and hepatitis A virus (HAV) [4].

NoVs are non-enveloped viruses belonging to the genus *Norovirus*, family Caliciviridae. These viruses are genetically variable and classified into 10 genogroups (GI-GX), among which NoV GI and GII represent those most frequently associated with human infections [5].

Hepatitis A virus (HAV) belongs to the genus *Hepatovirus*, family Picornaviridae. Human HAVs are divided into three genotypes (I, II and III) and seven subgenotypes (IA, IB, IC, IIA, IIB, IIIA, IIIB), of which the subtypes IA, IB and IIIA are responsible for the majority of human infections [6].

The role of marine gastropods in the accumulation and spread of human pathogenic viruses has been poorly investigated. An acute gastroenteritis outbreak associated with norovirus and sapovirus occurred in 2011 in Japan in subjects who had consumed raw sea snails (*Umbonium giganteum*) [7]. Further investigation pointed out the presence of norovirus and sapovirus in 59.3% of samples (*n* = 27) of sea snails collected in Japan during a period of five months [7].

Recently, it was evidenced that bivalve molluscs are able to accumulate fish viruses such as nervous necrosis virus (NNV). This virus belongs to the genus *Betanodavirus*, family Nodaviridae, and it is responsible for viral nervous necrosis, a disease that causes severe mortality outbreaks with nervous clinical signs in several marine fish species [8]. Betanodaviruses are divided into four genotypes: striped jack nervous necrosis virus (SJNNV), tiger puffer nervous necrosis virus (TPNNV), barfin flounder nervous necrosis virus (BFNNV) and redspotted grouper nervous necrosis virus (RGNNV). Furthermore, in the Mediterranean Basin, the presence of two reassortant strains, which developed from the RGNNV and SJNNV genotypes and are named RGNNV/SJNNV and SJNNV/RGNNV, has been reported [9]. Bivalve molluscs are able to accumulate and release infectious particles of this virus, and therefore they may represent a potential route of disease transmission to fish [10]. Moreover, a recent investigation pointed out the presence of this virus also in bivalve molluscs that were not close to fish farms of susceptible species, indicating their wide diffusion in the marine environment and a spatial and seasonal distribution related to the epidemiology of the disease [11].

Betanodaviruses are reported in a growing number of other invertebrate species such as cephalopods [12,13] and gastropods [14]. In particular, as regards gastropods, currently, they have only been reported once in the red-mouthed rock shell (*Stramonita haemastoma*) belonging to the Muricidae family [14].

As part of the project PO FEAMP ITALIA 2014–2020 “Pilot action for the sustainability of small-scale fishery in Emilia-Romagna Region”, an investigation was conducted to assess the presence of human and fish viruses in gastropods collected in the Adriatic Sea. In particular, the survey focused on *T. mutabilis*, in order to promote this important product for small-scale fisheries. Furthermore, the investigation on pathogenic viruses of aquatic animals was conducted to increase the surveillance of one of the most threatening pathogens for finfish species of the Mediterranean Sea and to evaluate the possible involvement of gastropods in the epidemiology of viral nervous necrosis.

## 2. Materials and Methods

### 2.1. Virological Investigation

A virological investigation was conducted on 34 batches of marine gastropods collected during the fishing season (October–May) from 2017 to 2021 on the Emilia Romagna coast. Gastropods were collected by fishermen using basket traps according to the small-scale fishery operations usually conducted in the Adriatic Sea (Regulation 854/2004/EC EUROPEAN COMMISSION, 2004). In particular, 27 batches of *T. mutabilis,* 6 batches of *Bolinus brandaris* and 1 batch of *Rapana venosa* were analyzed.

A molecular investigation was conducted to determine the absence/presence of 4 viruses: norovirus GI (NoV GI), norovirus GII (NoV GII), hepatitis A virus (HAV) and nervous necrosis virus (NNV).

Furthermore, for comparison purposes, a virological investigation on human pathogens was also conducted on 54 batches of Manila clam (*Ruditapes philippinarum*) collected in the north-western Adriatic Sea from 2017 to 2021.

The virological investigation was conducted on the digestive gland where microorganisms are known to persist [15,16,17,18]. For each sampling, digestive glands were collected from 10–15 specimens of *T. mutabilis*, 2–8 specimens of *Bolinus brandaris* and 3 specimens of *Rapana venosa.* Digestive gland samples were pooled together to reach an aliquot of at least 2 g.

Similarly, for clam samples, digestive glands were collected from 30 animals and pooled together to reach an aliquot of 2 g.

Digestive gland homogenates were subjected to viral RNA purification using the protease K method [15,16]. Briefly, the viral homogenates were digested with a solution of 20 mg/mL of protease K (Macherey-Nagel, Duren, Germany), incubating the samples at 37 °C for 1 h with agitation and then at 60 °C for 15 min. Then, the samples were centrifuged at 3000× *g* for 5 min to remove cellular debris. The obtained supernatants were used for RNA extraction using a commercially available kit (NucleoSpin RNA, Macherey-Nagel, Duren, Germany) following the manufacturer’s instructions. The investigation on the presence of viral RNA was conducted using specific and previously optimized protocols.

In particular, the presence of HAV was assessed through an RT-nested PCR assay targeting a VP1 fragment performed according to a method previously described [19].

The presence of noroviruses was evaluated using two real-time RT-PCR assays that enable a qualitative detection of NoV GI and NoV GII [20].

Regarding nervous necrosis virus, its presence was investigated in gastropods using an RT-nested PCR assay targeting the viral RNA1 segment [21]. Furthermore, an RT-nested PCR targeting the viral RNA2 was conducted on positive samples for genotyping [8]. Details of the used primers and probes are reported in Table 1. Positive and negative controls were run along with all reactions.

Amplicons obtained from these reactions were visualized through gel electrophoresis and then purified from agarose gel (Eurogold gel extraction kit, Euroclone, Milan, Italy) or from PCR products (Exosap, Affymetrix, Santa Clara, CA). Then, they were quantified through a fluorometer (Qubit 3.0, Life technologies, Carlsbad, CA) and sequenced with specific primers (Bio-Fab Research srl, Rome, Italy). The obtained sequences were manually corrected and analyzed through the online program Basic Local Alignment Search Tool (BLAST), available on the National Centre for Biotechnology Information site (NCBI, www.ncbi.nlm.nih.gov accessed on 2 February 2022), to confirm the viral identity.

To further characterize the HAV strain found in gastropods and bivalve molluscs, a phylogenetic analysis on the partial gene of the VP1 protein was conducted comparing the obtained sequences with those of the HAV reference strain HM-145 and of a selection of Italian HAV strains [30] available in the GenBank database (https://www.ncbi.nlm.nih.gov/genbank/ accessed on 2 February 2022). The alignment was conducted using the Clustal W method implemented in the BioEdit software (http://bioedit.software.informer.com/ accessed on 2 February 2022). Neighbor-joining phylogenetic analysis of the partial VP1 gene was performed with MEGA X software (www.megasoftware.net accessed on 2 February 2022). Bootstrap analysis was carried out on 1000 replicates. The genotyping of NoVs was conducted using the Norovirus Typing Tool Version 2.0 [31] by analyzing a fragment of the RNA-dependent RNA polymerase (RdRp).

Regarding NNV strains found in gastropods, which have a bi-segmented RNA genome, a phylogenetic analysis was performed using both RNA1 and RNA2 gene fragments of the virus. The sequences of the detected viruses were aligned and compared with NNV sequences identified in both marine vertebrates and invertebrates as well as with betanodavirus reference strains available in GenBank (https://www.ncbi.nlm.nih.gov/genbank/ accessed on 26 April 2022) using Clustal W implemented in the BioEdit software (http://bioedit.software.informer.com/ accessed on 26 April 2022). The phylogenetic analysis was conducted using the maximum likelihood algorithm applied to partial RNA1 and RNA2 sequences with MEGA X software (www.megasoftware.net accessed on 26 April 2022). Bootstrap analysis was carried out on 1000 replicates.

### 2.2. Histology

Fourteen batches of *T. mutabilis*, including the three positives for NNV, were employed for histological investigation. Gastropods were immersed in an anesthetic overdosing solution (ethanol 1%) [32]. Then, the animals were placed in Bouin’s fixative solution (PanReac AppliChem ITW Reagents, Castellar del Valees, Spain), taking advantage of its mild decalcifying effect and producing higher preservation of tissues [33]. Before processing, the animals were immersed in a decalcifying solution (Osteosens, Histo-Line, Milan, Italy) for 24 h and then longitudinally cut with a scalpel. Subsequently, they were routinely processed (embedding centre, Histo-line, Milan, Italy). From the formalin-fixed paraffin-embedded biocassettes, 3 µm-thick sections were obtained (rotative microtome, Leica Microsistem, Milan, Italy) and stained with hematoxylin–eosin (H&E, Histoline, Milan, Italy). The histological investigation included the evaluation of all animal tissues in general and focused on the identification of the ganglia, searching for potential changes (i.e., vacuolar degeneration, necrosis) which are reported in the nervous tissue and eyes of finfish affected by VNN.

## 3. Results

### 3.1. Human Virus Detection and Genotyping

Hepatitis A virus was detected in 1 sample (collected in 2020) out of 27 of *T. mutabilis* (3.7% of *T. mutabilis* samples and 2.9% of all gastropods). Furthermore, 3 bivalve mollusc samples (collected in 2017 and 2019) out of 54 resulted in being positive for HAV, representing 5.6% of the *R. philippinarum* samples (Table 2). Sequences obtained from gastropod and bivalve mollusc samples were submitted to the GenBank database (OP204623-OP204626).

**Figure 1:**
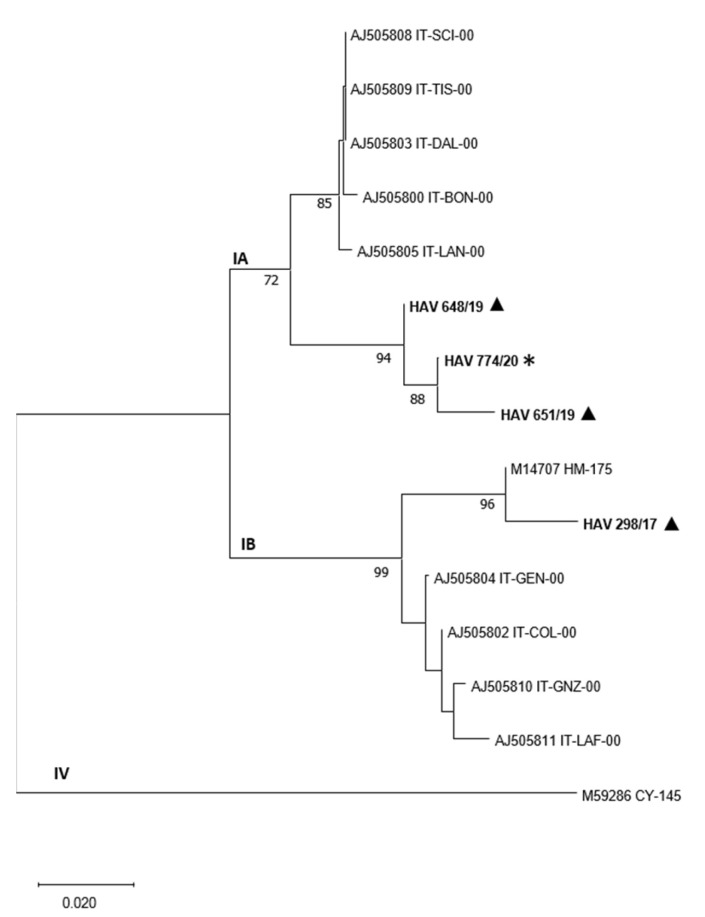
Neighbor-joining phylogenetic tree based on the partial VP1 nucleotide sequences of HAV. Sequences obtained in this study including those from gastropods (asterisk) and those from bivalve molluscs (triangle) are in bold. Sequences retrieved from GenBank are reported with the isolate name and the accession number. Bootstrap values > 70% are shown. Branch lengths are scaled according to the number of nucleotide substitutions per site. The scale bar is reported.

The viral strain of HAV detected in gastropods belonged to subtype IA (Figure 1), whereas in bivalve molluscs, the presence of both genotypes IA (samples collected in 2019) and IB (sample collected in 2017) was found.

Regarding NoV, its presence was not detected in any of the tested gastropod samples. Contrariwise, the presence of NoV was shown in 19 out of 54 clam samples (35.2%; Table 2). In particular, 17 clam samples were contaminated with NoV GII, and 2 were contaminated with NoV GI. Furthermore, one sample showed co-contamination with HAV and NoV GII.

### 3.2. Fish Virus Detection and Genotyping

Regarding fish viruses, NNV was detected in 9 samples out of 34 (26.5%), specifically in 4 specimens of *B. brandaris* and 5 samples of *T. mutabilis* (Table 2). Sequences obtained from gastropod samples were submitted to the GenBank database (OP234311- OP234319 and OP251131-OP251137).

**Figure 2:**
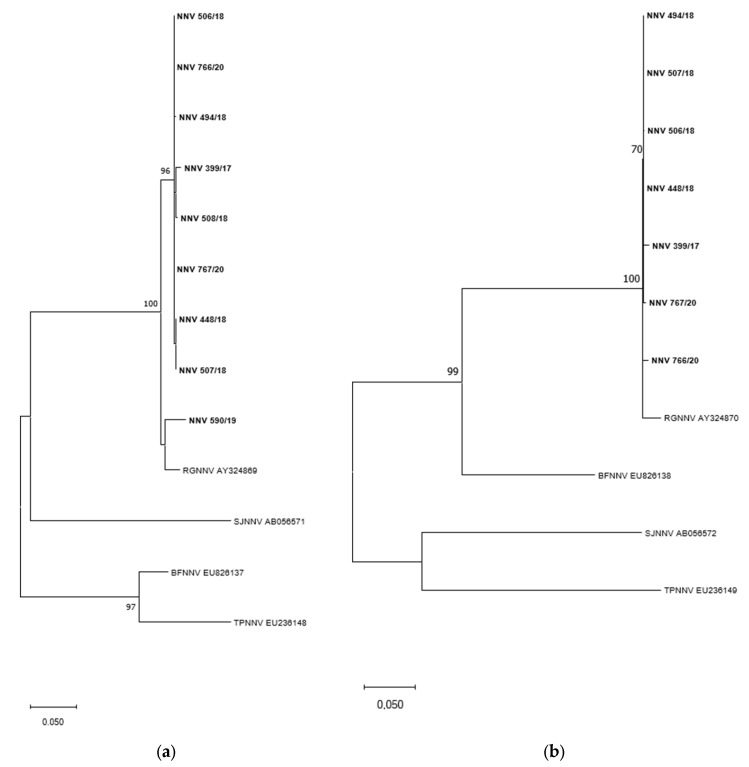
Maximum likelihood phylogenetic tree based on the partial RNA1 (**a**) and RNA2 (**b**) nucleotide sequences of NNV. Sequences obtained in this study are in bold. Sequences retrieved from GenBank are reported with the genotype name and the accession number. Bootstrap values >70% are shown. Branch lengths are scaled according to the number of nucleotide substitutions per site. The scale bar is reported.

The analysis of NNVs’ genome allowed us to determine the viral genotypes. Both RNA1 and RNA2 sequences of all detected NNVs clustered with the RGNNV reference strain (Figure 2), showing that they belong to the RGNNV genotype.

### 3.3. Histology

**Figure 3:**
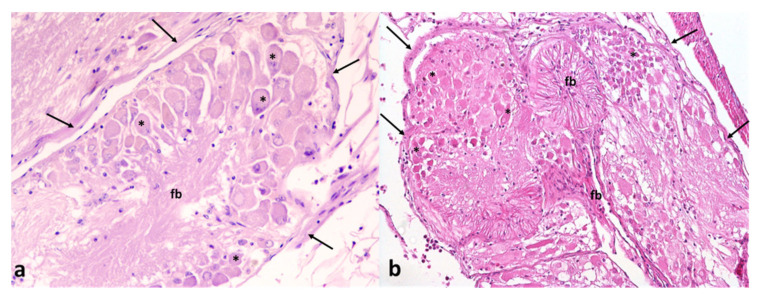
Nerve ganglia of *T. mutabilis*. Ganglia are lined by a rim of connective tissue (arrows) and are composed of pyrenophores (asterisks) and fiber bundles (fb). No signs of vacuolar degeneration or necrosis were present in (**a**) NNV-negative and (**b**) NNV-positive animals. Hematoxylin and eosin, magnification ×200 and ×100.

The examined animal tissues of *T. mutabilis* did not show histological changes. In particular, regarding ganglia, no degenerative changes (i.e., vacuolar degeneration or necrosis) were detected in neurons and in fiber bundles of NNV-positive and -negative animals (Figure 3a,b).

**Figure 4:**
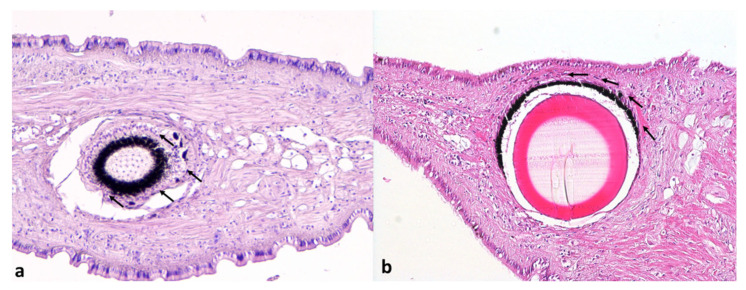
Eyes of *T. mutabilis*: (**a**) NNV-negative and (**b**) NNV-positive animals. The photoreceptors and basal retinal neurons (arrows) did not show signs of degeneration. Hematoxylin and eosin, magnification ×100.

Furthermore, no histological changes affecting the photoreceptors and the basal retinal neurons of the eyes were detected (Figure 4a,b).

## 4. Discussion

The present virological investigation pointed out the occurrence of viruses, including both human and fish viruses, in two species of marine gastropods.

The presence of human viruses in aquatic gastropods has been poorly investigated hitherto; however, some studies pointed out the presence of human enteric viruses in freshwater and marine gastropods [7,34]. In particular, thus far, norovirus, sapovirus and adenovirus have been detected, whereas, to the best of our knowledge, hepatitis A virus, frequently associated with bivalve mollusc contamination [4], has never been searched for and found in aquatic gastropods. In the present study, hepatitis A virus was detected in one *T. mutabilis* batch representing 2.9% of all tested gastropod batches. In the bivalve molluscs screened in this study for comparison, 5.6% of the samples were positive for HAV. These results are consistent with previous monitoring programs on HAV diffusion carried out in bivalve molluscs collected in Italy, which showed very low prevalence levels (0.9–6%) or a complete absence of this virus [35,36,37]. A recent study investigating the presence of HAV in mussels collected in an urbanized area, not intended for consumption, showed a slightly higher prevalence (7%) [11]. The viral strain of HAV detected in bivalve molluscs belonged to subtype IA. Genotype I is considered the most widespread all around the world, and notably, the IA subtype is more diffuse than the IB subtype. The HAV IA subtype has been frequently associated with food-borne human infection due to the consumption of raw seafood in Southern Italy [30]. In bivalve molluscs, instead, the presence of both genotypes IA and IB was found.

On the contrary, no presence of NoV was detected in any of the tested gastropod samples, whereas the presence of this virus was observed in 37.4% of clams, with a higher prevalence of NoV GII (85% of the positive samples) than NoV GI (15% of the positive samples). The result obtained in clams is in accordance with previous investigations conducted on bivalve molluscs sampled in the Adriatic Sea, which have already shown high prevalence values for NoV (22–51.4%) [11,35,38]. Similar percentages of positivity in bivalve molluscs and a greater presence of NoV GII have already been described in surveys conducted in other Italian regions (NoV 14.2–51.5%; GII 12.2–49.4%; GI 1.6–26.4%) [36,37]. NoV GI represents the genogroup more frequently associated with human infections due to the consumption of contaminated bivalve molluscs and water [39], while NoV GII represents the genogroup more frequently detected worldwide [40].

Despite the fact that gastropods are not filter feeders like bivalve molluscs, the present study and a few previous reports [7,34] showed their ability to bioaccumulate human enteric viruses, posing a potential risk for consumers. However, thus far, only one acute gastroenteritis outbreak associated with gastropod consumption has been reported [7], showing a lower risk associated with gastropods than with bivalve molluscs. Accordingly, our study pointed out lower contamination in the tested gastropods compared to the bivalve molluscs. The reduced risk could be the result of several different reasons such as a lower accumulation ability, lower exposure to contaminated water, lower human consumption and a different method of consumption (cooked vs. raw). Further studies focusing on viruses in gastropods are needed to improve our knowledge and to minimize the risk for consumers.

Regarding fish viruses, this study pointed out a frequent presence of NNV in gastropods of both species *T. mutabilis* and *B. brandaris*. The analysis of NNVs’ genome showed that all the detected NNVs clustered with the RGNNV genotype, the most widespread in the Mediterranean Sea; no reassortant strains were detected in the gastropods.

Despite the fact that RGNNV is a well-known finfish pathogen, its presence in invertebrates has been frequently reported too. In particular, it has been identified in several species of bivalve molluscs [8,11,41], cephalopods [12,13,42] and crustacea [13,41,43]. The presence of RGNNV in invertebrates has not been associated, thus far, with any pathological finding. However, other viruses of the family Nodaviridae are well-known pathogens for invertebrate species (mainly crustacea), and a virus of this family able to infect both vertebrates and invertebrates has been described [44]. Furthermore, despite the fact that viral replication has never been reported for RGNNV in invertebrates, the ability of bivalve molluscs to accumulate and release infectious viral particles has been experimentally demonstrated [10].

Recently, RGNNV presence was also reported in one gastropod in Greece [14]; however, its potential pathological role has not been investigated.

The presence of RGNNV in gastropods in our study was quite high, with 26.5% of the batches being positive for the virus. Similar contamination levels have already been observed in bivalve molluscs. In particular, a study conducted on commercial bivalve molluscs showed an NNV contamination rate of 26.3% of the tested samples including clams, mussels and oysters [8], with higher contamination percentages in clams (42.1%) and oysters (31.6%) and less involvement in mussels (5.3%). However, a study focused on mussels was able to point out an NNV contamination rate of 36.7% of the tested samples, showing how, in the presence of contaminated water, all species of bivalve molluscs can be subjected to frequent contamination [11]. In this regard, it seems that also marine gastropods may have a high bioaccumulation potential for viruses that are present in the water.

Viruses from a number of families have been reported in gastropods such as Herpesvirusae, Bacilladnaviridae, Circoviridae, Reoviridae, Picornaviridae, Caliciviridae, Paramyxoviridae and Rhabdoviridae [45,46]; however, their role and pathogenic potential have not been investigated, apart from Haliotid herpesvirus-1 (HaHV-1), the well-known cause of abalone viral ganglioneuritis [47].

Nodaviruses are neurotropic viruses generally associated with necrosis and vacuolar degeneration in both vertebrates and invertebrates [44,48]. In particular, in affected finfish, RGNNV is associated with extensive degenerative lesions of the central nervous system (CNS) and retina, visible as multifocal vacuolization in the internal and external layers of the retina, optic nerve and brain, as well as pyknosis and karyorrhexis in the whole CNS [9,48].

The histological investigation, focused on the evaluation of *T. mutabilis* neurons and nerve bundles, constituting the visceral ganglia, and those belonging to the retina, did not reveal signs of alteration. In particular, the typical pathological signs were not present. The absence of degenerative changes in the neurons of NNV-positive gastropods suggests that these animals are capable of accumulating the virus without displaying signs of cell suffering. Despite the fact that we cannot exclude viral replication activity in the investigated tissues, RGNNV does not seem to induce pathologic effects in NNV-positive gastropods.

## 5. Conclusions

The presence of both human and fish viruses was found in gastropods. In particular, RGNNV was found in a reasonably high number of batches, similar to what has already been shown in bivalve molluscs. However, the histological investigation did not show any alterations in the nervous tissue, which is usually targeted by RGNNV in susceptible hosts, suggesting a low pathogenicity, or lack thereof, of this virus in gastropods. Furthermore, HAV was detected in one batch of *T. mutabilis,* confirming the ability of the gastropod to accumulate human viruses. Despite that, the low presence of these viruses in gastropods, compared to bivalve molluscs, along with the typical habit to serve this food well-cooked, suggests a low risk of food-borne viral infections for gastropod consumers.

## Figures and Tables

**Table 1:** Details of primers and probes used in this study.

Virus	Target Region	Primer Name	Sequence	Reference
HAV RT-nested PCR	VP1	AV1	5′-GGAAATGTCTCAGGTACTTTCTTTG-3′	[19]
		AV2	5′-GTTTTGCTCCTCTTTATCATGCTATG-3′	
		AV3	5′-TCCTCAATTGTTGTGATAGC-3′	
NoV RT real-time PCR	RdRp	NVGG1p	5′-FAM-TGGACAGGAGAYCGCRATCT-3′TAMRA	[22]
		NV1LCR	5′-CCTTAGACGCCATCATCATTTAC-3′	
		QNIF4	5′-CGTGGATGCGNTTCCAT-3′	[23]
		QNIFS	5′-FAM-AGCACGTGGGAGGGCGATCG-3′TAMRA	
		COG2R	5′-TCGACGCCATCTTCATTCACA-3′	[24]
		QNIF2	5′-ATGTTCAGRTGGATGAGRTTCTCWGA-3′	[23]
NoV RT-nested PCR	RdRp	JV12	5′-ATACCACTATGATGCAGATTA-3′	[25]
		JV13	5′-TCATCATCACCATAGAAAGAG-3′	
		NoVG1	5′-TCNGAAATGGATGTTGG-3′	[26]
		NoVG2	5′-AGCCAGTGGGCGATGGAATTC-3′	[27]
NNV RT-nested PCR	RNA1	VNNV5	5′-GTTGAGGATTATCGCCAACG-3′	[21]
		VNNV6	5′-ACCGGCGAACAGTATCTGAC-3′	
		VNNV7	5′-CACTACCGTGTTGCTG-3′	
NNV RT-nested PCR	RNA2	S6	5′-ATGGTACGCAAAGGTGATAAGAAA-3′	[28]
		S7	5′-GTTTTCCGAGTCAACACGGGT-3′	
		F2	5′-CGTGTCAGTCATGTGTCGCT-3′	[29]
		R3	5′-CGAGTCAACACGGGTGAAGA-3′	

**Table 2:** Results of the virological investigation.

	Gastropods			Bivalve Molluscs
	*T. mutabilis*	*B. brandaris*	*R. venosa*	*R. philippinarum*
HAV	1 (3.7%)	0	0	3 (5.6%)
NoV	0	0	0	19 (35.2%)
NNV	5 (18.5%)	4 (66.7%)	0	n.d.

## Data Availability

Sequences obtained in this study have been submitted to the GenBank repository available at https://www.ncbi.nlm.nih.gov/genbank/, accessed on 10 August 2022.
